# Identification of a novel autoantigen eukaryotic initiation factor 3 associated with polymyositis

**DOI:** 10.1093/rheumatology/kez406

**Published:** 2019-09-17

**Authors:** Zoe Betteridge, Hector Chinoy, Jiri Vencovsky, John Winer, Kiran Putchakayala, Pauline Ho, Ingrid Lundberg, Katalin Danko, Robert Cooper, Neil McHugh

**Affiliations:** 1 Pharmacy and Pharmacology, University of Bath, Bath; 2 National Institute for Health Research, Manchester University NHS Foundation Trust, The University of Manchester, Manchester; 3 Department of Rheumatology, Salford Royal NHS Foundation Trust, Manchester Academic Health Science Centre, Salford, UK; 4 Rheumatology, Charles University, Prague, Czech Republic; 5 University Hospital Birmingham, Queen Elizabeth Hospital, Birmingham; 6 Department of Rheumatology, Leighton Hospital, Crewe; 7 Department of Rheumatology, Manchester Royal Infirmary, Manchester, UK; 8 Division of Rheumatology, Department of Medicine, Karolinska Institutet, Karolinska University Hospital, Stockholm, Sweden; 9 Immunology, Department of Internal Medicine, University of Debrecen, Debrecen, Hungary; 10 Department of Musculoskeletal Biology II, University of Liverpool, Liverpool, UK

**Keywords:** autoantibodies, autoantigens, myositis

## Abstract

**Objectives:**

To describe the prevalence and clinical associations of autoantibodies to a novel autoantigen, eukaryotic initiation factor 3 (eIF3), detected in idiopathic inflammatory myositis.

**Methods:**

Sera or plasma from 678 PM patients were analysed for autoantigen specificity by radio-labelled protein immunoprecipitation (IPP). Samples immunoprecipitating the same novel autoantigens were further analysed by indirect immunofluorescence and IPP using pre-depleted cell extracts. The autoantigen was identified through a combination of IPP and MALDI-TOF mass spectrometry, and confirmed using commercial antibodies and IPP-western blots. Additional samples from patients with DM (668), DM-overlap (80), PM-overlap (191), systemic sclerosis (150), systemic lupus erythematosus (200), Sjogren’s syndrome (40), rheumatoid arthritis (50) and healthy controls (150) were serotyped by IPP as disease or healthy controls.

**Results:**

IPP revealed a novel pattern in three PM patients (0.44%) that was not found in disease-specific or healthy control sera. Indirect immunofluorescence demonstrated a fine cytoplasmic speckled pattern for all positive patients. Mass spectrometry analysis of the protein complex identified the target autoantigen as eIF3, a cytoplasmic complex with a role in the initiation of translation. Findings were confirmed by IPP-Western blotting. The three anti-eIF3-positive patients had no history of malignancy or interstitial lung disease, and had a favourable response to treatment.

**Conclusion:**

We report a novel autoantibody in 0.44% of PM patients directed against a cytoplasmic complex of proteins identified as eIF3. Although our findings need further confirmation, anti-eIF3 appears to correlate with a good prognosis and a favourable response to treatment.

**Rheumatology key messages**

- Autoantibodies to eIF3 are present in 0.44% of adult PM patients.
- Autoantibodies to eIF3 may identify a form of polymyositis more responsive to immunosuppressive treatment.

## Introduction

PM and DM are heterogeneous conditions characterized by proximal muscle inflammation and weakness, characteristic skin lesions and extramuscular features, including interstitial lung disease (ILD) and cancer [1, 2]. There is strong evidence for a role of autoimmunity in disease pathogenesis, with up to 70% of patients developing either myositis-specific or myositis-associated autoantibodies (MSAs/MAAs) [3]. These autoantibodies target specific proteins in the cell involved in gene transcription, protein translocation and anti-viral responses. The MSAs/MAAs help define more homogeneous clinical subsets of myositis [4], thus aiding in the prediction of disease course and treatment outcomes. The MAAs are commonly found in patients with overlap features of other connective tissue diseases, whereas MSAs are found almost exclusively in PM and DM [4], and here correlate strongly with HLA genotypes and clinical phenotypes [3].

In PM, the commonest MSAs are directed against the cytoplasmic aminoacyl-transfer RNA synthetases (ASAs), and are associated with a distinct phenotype termed the anti-synthetase syndrome. To date, eight ASAs have been fully described: Jo-1 is the most common, occurring in ∼20% of myositis patients, with the remaining ASAs (anti-PL12, anti-PL7, anti-OJ, anti-EJ, anti-KS, anti-Zo and anti-Ha) each occurring in <1–5% of PM patients [3]. Recently, the concept of immune-mediated necrotizing myopathies has gained recognition and together with the anti-synthetase syndrome accounts for a majority of patients previously classified as PM [5]. Autoantibodies associated with immune-mediated necrotizing myopathies include anti-signal recognition particle (SRP) [6] and anti-3-hydroxy-3-methylglutaryl-coenzyme A reductase (HMGCR) [7]. Herein, we describe the identification of a further myositis autoantibody, anti-eukaryotic initiation factor 3 (eIF3) in three patients with PM.

## Methods

### Patients and sera

Clinical data and serum/plasma samples from 678 adult PM patients recruited to either the UKMYONET or EuroMyositis cohorts were available for analysis [4]. All patients had probable or definite myositis according to the Bohan and Peter diagnostic criteria [1, 2]. A standardized pro forma was used throughout to collect demographic and clinical case details [8]. The disease control population consisted of 668 DM, 80 DM-overlap and 191 PM-overlap patients [1, 2], 150 patients with systemic sclerosis [9], 200 patients with SLE [10], 40 patients with SS [11], 50 patients with rheumatoid arthritis [12] and 100 otherwise healthy controls. Written consent to participate and to provide biological samples was obtained from all subjects according to the Declaration of Helsinki, under the local ethical committee regulations of each participating centre.

### Protein immunoprecipitation using [^35^S]-methionine

10 µl sera or plasma was mixed with 2 mg protein-A-Sepharose beads (Sigma, Gillingham, UK) in protein immunoprecipitation (IPP) buffer (10 mM Tris-Cl pH 8.0, 500 mM NaCl, 0.1% v/v Igepal) at room temperature for 30 min. Beads were washed in IPP buffer prior to the addition of 120 µl [^35^S]-methionine labelled K562 cell extract (prepared from 15 × 10^6^ cells/ml). Samples were mixed at 4°C for 2 h. Beads were washed in IPP buffer and TBS buffer (10 mM Tris-Cl pH 7.4, 150 mM NaCl) before re-suspension in Sample Buffer (Sigma, Gillingham, UK). After heating, proteins were fractionated by 9% SDS–PAGE and analysed by autoradiography.

### Indirect immunofluorescence

Indirect immunofluorescence was performed on HEp-2 cells (Immunoconcepts, USA) using IgG fluorescein-labelled anti-Human immunoglobulin (Sigma, Gillingham, UK).

### IPP-Mass spectrometry (MS)

IPP was completed using 40 µl plasma, 2 mg protein-A-Sepharose beads and 5 mM bis-(sulphosuccinimidyl)-suberate (Perbio, UK). Beads were incubated in 4 ml K562 cell extract (prepared from 28 × 10^6^ cells/ml) for 4 h at 4°C. Samples were re-suspended in Sample Buffer. Proteins were heated, fractionated by 10% SDS–PAGE and stained with Imperial Protein Stain (Perbio, UK). Unique bands were prepared for MALDI-TOF MS at the University of the West of England. Database matching using the ProteinLynx software required peptide coverage of over 20% with matching of the major theoretical and experimental peptide peaks.

### IPP-blotting

IPP was completed using 40 µl plasma/serum (cases 1, 2 or 3, normal control serum or serum containing known autoantibodies), 2 mg protein-A-Sepharose beads and 5 mM bis-(sulphosuccinimidyl)-suberate (Sigma, Gillingham, UK). Beads were incubated in 1 ml K562 cell extract (prepared from 28 × 10^6^ cells/ml) for 3 h at 4°C. Samples were re-suspended in sample buffer (Sigma, Gillingham, UK), heated and fractionated by 10% SDS–PAGE. Immunoprecipitates were transferred to nitrocellulose and probed with rabbit polyclonal anti-eIF3D antibody (1 : 500 dilution) (Sigma, Gillingham, UK) for 90 min. Bands were detected using alkaline phosphatase conjugated goat anti-rabbit IgG (1 : 20000 dilution) (Sigma, Gillingham, UK) antibody and BCIP/NBT liquid substrate solution (Sigma, Gillingham, UK).

## Results

### Serotyping of PM patients

**Fig. 1:**
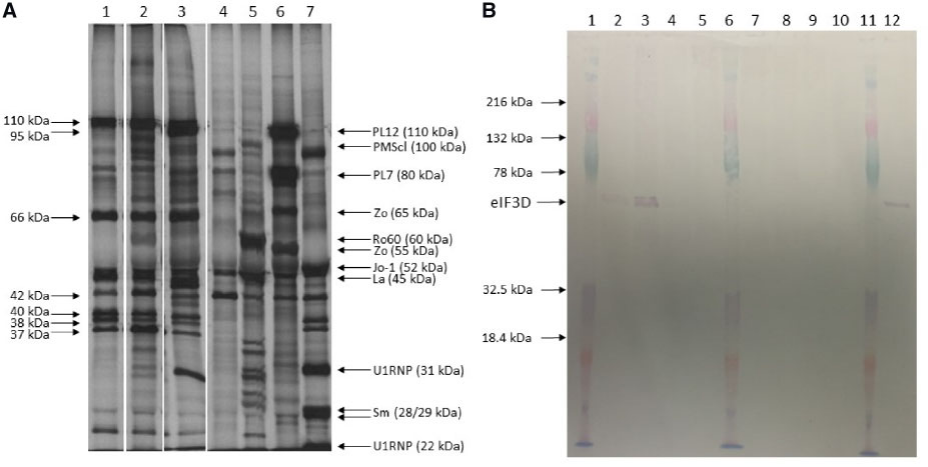
Protein immunoprecipitation and immunoprecipitation/blot using anti-EIF3 sera from three cases and controls **(A)** Autoradiogram of 10% SDS–PAGE of immunoprecipitates using case 1–3 serum/plasma (lanes 1–3), healthy control serum (lane 4), control serum containing autoantibodies to Ro-60, La and PMScl (lane 5), PL7, PL12 and Zo (lane 6) and snRNP and Jo-1 (lane 7) using [^35^S] methionine-labelled cell extract. Arrows indicate apparent molecular weights of autoantigens immunoprecipitated by some or all case samples. **(B)** IPP-blot of eIF3 autoantigens. Antigens were immunoprecipitated using case sera/plasma or controls. Lanes 1, 6 and 11: molecular weight marker; lane 2: case 1 plasma; lane 3: case 2 plasma; lanes 4 and 5: IPP eIF3 negative PM serum; lane 7: healthy control serum; lane 8: serum containing anti-PMScl, La and Ro-60; lane 9: serum containing anti-snRNP and anti-Jo-1; lane 10: serum containing anti-PL7 and anti-PL12; lane 12: case 3 serum. Immunoprecipitates were separated on SDS–PAGE and transferred to a nitrocellulose membrane. Membranes were probed with commercial anti-eIF3D. Bands corresponding to eIF3D and the molecular weight markers are indicated.

An IPP screen was completed on 678 PM patients. The results showed the presence of the same IPP pattern in three patients (cases 1–3). Samples from each patient immunoprecipitated antigens at ∼25, 37, 39, 40, 42, 66, 95 and 110 kDa (Fig. 1A, Lanes 1–3), and these did not correspond to any known autoantigen. None of these patients immunoprecipitated any other known myositis-specific or associated autoantigen. This novel pattern was not seen in any immuno-precipitations using sera/plasma from DM, DM-overlap or PM-overlap patients, or any of the connective tissue disease or healthy controls. Immunofluorescence of HEp2 cells using cases 1–3 resulted in either a negative or weak, fine speckled ANA, and a fine cytoplasmic speckle (data not shown). Overall the prevalence of anti-eIF3 in PM was 0.4%.

### Identification of the eIF3 antigens

Non-radiolabelled IPP demonstrated the presence of 37, 38, 40, 42, 66, 95 and 110 kDa bands using case 1 plasma on two separate occasions (data not shown). MALDI-TOF MS and Swiss-Prot analysis of the peptide fingerprints from these bands corresponded to Eukaryotic Initiation Factor 3 subunits (eIF3G, eIF3I, eIF3H, eIF3E/F eIF3L/D eIF3B and eIF3A respectively).

The autoantigen target was confirmed by IPP-blot using commercial eIF3D protein. Immunoprecipitations were completed using case 1, 2 or 3 plasma/serum, healthy control serum or control samples containing known autoantibodies. When the immunoprecipitates were transferred to nitrocellulose and probed with commercial anti-eIF3D, strong bands, at the correct molecular weight for eIF3D, were present with immunoprecipitates from cases 1, 2 and 3 but not from any of the disease or healthy control samples (Fig. 1B). Overall these results show that the novel precipitation pattern seen with the three PM patients is due to autoantibodies directed against eIF3.

### Clinical data

Clinical analysis of the anti-eIF3 patients demonstrated two of three patients to be female, with a median age at myositis onset for all three patients of 55 (range 53–58). As well as fulfilling Bohan and Peter criteria for PM, all three patients fulfilled EULAR-ACR classification criteria [13] of at least probable idiopathic inflammatory myopathy, with no evidence of rash or overlap features. None of the patients had cancer-associated myositis or interstitial lung disease. Other than mild Raynaud’s phenomenon in one patient and arthritis in another, there were no features of anti-synthetase syndrome. All anti-eIF3 positive patients had highly elevated creatine kinase (CK) levels (ranging 17–65 times the local cut-off values) and a significant pattern of proximal muscle weakness at presentation, with no evidence of distal muscle weakness. Of interest, two patients had a prior history of cardiovascular disease (heart conduction defect and a history of myocardial infarction). Although one of the three patients has a history of statin exposure, there were no features of immune-mediated necrotising myopathy on muscle biopsy (Table 1) and all three were negative for anti-HMGCR autoantibodies (data not shown). All of the anti-eIF3 patients were treated with prednisolone with two patients also being given azathioprine (Table 1). The small sample size precludes formal statistical analysis of clinical associations. However, all three anti-eIF3 positive patients responded well to treatment, being able to taper off prednisolone completely and lacked severe organ complications, suggesting it is feasible that anti-eIF3 autoantibodies are a marker of a favourable outcome.

**Table 1:** Clinical manifestations of anti-eIF3 positive patients and comparison with the anti-eIF3 negative PM cohort

	Case 1	Case 2	Case 3	EIF3 negative
AOO	58	53	55	Median = 55 (IQR: 42.00–64.00)
	Median = 55 (IQR: 54.00–56.50)			
Gender	Female	Female	Male	213 M / 455 F (32: 68% )
CAM	No	No	No	49/654 (7.5%)
ILD	No	No	No	197/655 (30.1%)
Raised CK	(8815 U/L)	(5940 U/L)	(2754^a^ U/L)	598/631 (94.8%)
Proximal weakness	Yes	Yes	Yes	494/511 (96.7%)
Weight loss	No	Yes	No	Data not collected
Arthritis	No	No	Yes	269/632 (42.6%)
EMG	Positive	Positive	Negative	309/420 (73.6%)
Muscle MRI	Widespread oedema most marked within adductors and rectus femori	Patchy high signal on STIR sequences throughout thigh muscles	Diffuse strong oedema thigh muscles and perifascial spaces	
Muscle biopsy	Positive – further details not available	Scant endomysial inflammation. Perivascular B lymphocytes. HLA Class I expression on regenerating fibres^b^	Large predominantly perimysial inflammatory infiltrate also endomysial. HLA Class I over-expression on muscle fibres	Data not collected
Raynaud’s phenomenon	No	No	Mild	203/487 (41.6%)
Cardiac involvement	None	Heart conduction defect	Myocardial Infarction (age 47)	34/400 (8.5%)
Treatment	Prednisolone and azathioprine	1 mg/kg prednisolone and azathioprine	40 mg/day prednisolone	Data not collected
Response to treatment	Good: prednisolone discontinued after 1 yr	Good: prednisolone discontinued. Sustained remission on 50 mg azathioprine	Good: prednisolone discontinued after 2 yr	Data not collected

a CK of 46 µkat/l converted to units/l using www.amamanualofstyle.com/page/si-conversion-calculator.

b Muscle biopsy taken two weeks after commencement of steroids.

AOO: age of onset; CAM: cancer associated myositis; CK: creatine kinase; F: Female; ILD: interstitial lung disease; IQR: interquartile range; M: Male; STIR, short-TI inversion recovery.

## Discussion

MSAs identify homogeneous clinical subsets within the IIM spectrum [4]. The identification of a novel MSA is therefore of interest, and may define a further clinical sub-group that predicts the nature of subsequent disease. Herein we describe the first report of autoantibodies directed against eIF3 in three patients with clinical features of PM. Anti-eIF3 autoantibodies are rare but occur at a similar frequency, in this predominantly Caucasian cohort, as a number of the non-Jo-1 ASAs. None of our eIF3 patients had features of CTD overlap, cancer-associated myositis or interstitial lung disease, and all demonstrated a good response to treatment. Therefore, patients with this autoantibody appear to have a favourable prognosis compared with some other myositis subsets, although larger numbers are needed to confirm this notion. Furthermore, the combination of clinical features towards a milder end of the myositis disease spectrum could indicate that anti-eIF3 patients are less likely to be referred to myositis clinics, and thus may be under-represented in myositis cohort studies.

As demonstrated by the IPP results, mammalian eIF3 is a multi-subunit complex with an apparent molecular weight of ∼650 kDa, consisting of 13 non-identical subunits that range in size from 25 to 170 kDa [14]. Although our study has demonstrated the presence of autoantibodies to eIF3 in PM patients, it remains to be determined whether these autoantibodies are targeted towards the entire complex or a particular subunit. The eIF3 complex is mainly located in the cytoplasm, which is consistent with the cytoplasmic staining pattern seen on indirect immunofluorescence with all anti-eIF3 positive sera. Mammalian eIF3 has been reported to have multiple functions, but primarily acts to ensure assembly of the 43S pre-initiation complex by direct recruitment of the 40S ribosomal subunit [15]; therefore, along with the other PM associated autoantigens (SRP and the ASAs), eIF3 has an important role in protein translation.

Of interest, studies have shown a further role for eIF3F in skeletal muscle regulation. Skeletal muscle atrophy is characterized by a decrease in the size of pre-existing muscle fibres and is seen in a number of physiological and pathological settings including myositis. In several models of skeletal muscle atrophy, the E3 ubiquitin ligase (Atrogin/MAFbx) is up-regulated. Increased levels of the ligase target eIF3F for ubiquitination and degradation by the proteosome, leading to rapid atrophy [16, 17]. Furthermore, in mouse models, eIF3f depletion impedes embryonic development, reduces adult skeletal muscle mass and amplifies muscle loss during disuse [18]. All of the anti-eIF3 positive patients identified in this cohort had clinical symptoms of muscle weakness and whilst no details regarding muscle atrophy was available for our patients, these findings indicate a potential pathogenic link between the role of eIF3 in skeletal muscle atrophy and the presence of eIF3 autoantibodies. It would be of interest to investigate whether the anti-eIF3 positive patients have evidence of muscle atrophy and whether the eIF3F subunit is specifically targeted by the anti-eIF3 autoantibodies.

In conclusion, we have described a novel autoantibody, anti-eIF3 autoantibody, in a small number of PM patients who are negative for all other MSA/MAAs. Whilst the numbers are small, the presence of the autoantibody appears to associate with a favourable prognosis, with an absence of malignancy and ILD, and a good response to immunosuppression. The eIF3 protein shares similar features to other PM autoantigens, in that it plays a key role in protein synthesis. Additionally, the autoantibody targeting of a protein complex involved in myotube integrity may provide further insight into the pathogenesis of myositis.
